# Elevated Terminal C5b-9 Complement Complex 10 Weeks Post Kidney Transplantation Was Associated With Reduced Long-Term Patient and Kidney Graft Survival

**DOI:** 10.3389/fimmu.2021.738927

**Published:** 2021-10-25

**Authors:** Bartlomiej J. Witczak, Søren E. Pischke, Anna V. Reisæter, Karsten Midtvedt, Judith K. Ludviksen, Kristian Heldal, Trond Jenssen, Anders Hartmann, Anders Åsberg, Tom E. Mollnes

**Affiliations:** ^1^ Department of Nephrology, Akershus University Hospital, Lørenskog, Norway; ^2^ Department of Immunology, Oslo University Hospital, University of Oslo, Oslo, Norway; ^3^ Department of Anaesthesiology, Oslo University Hospital–Rikshospitalet, Oslo, Norway; ^4^ Department of Transplantation Medicine, Oslo University Hospital–Rikshospitalet, Oslo, Norway; ^5^ Norwegian Renal Registry, Oslo University Hospital–Rikshospitalet, Oslo, Norway; ^6^ Research Laboratory, Nordland Hospital, Bodø, Norway; ^7^ Institute of Clinical Medicine, University of Oslo, Oslo, Norway; ^8^ Department of Pharmacy, University of Oslo, Oslo, Norway; ^9^ Faculty of Health Sciences, KG Jebsen Thrombosis Research and Expertise Center (TREC), University of Tromsø, Tromsø, Norway; ^10^ Centre of Molecular Inflammation Research, Norwegian University of Science and Technology, Trondheim, Norway

**Keywords:** biomarker, complement, kidney transplantation, graft survival, patient survival

## Abstract

**Background:**

The major reason for graft loss is chronic tissue damage, as interstitial fibrosis and tubular atrophy (IF/TA), where complement activation may serve as a mediator. The association of complement activation in a stable phase early after kidney transplantation with long-term outcomes is unexplored.

**Methods:**

We examined plasma terminal C5b-9 complement complex (TCC) 10 weeks posttransplant in 900 patients receiving a kidney between 2007 and 2012. Clinical outcomes were assessed after a median observation time of 9.3 years [interquartile range (IQR) 7.5–10.6].

**Results:**

Elevated TCC plasma values (≥0.7 CAU/ml) were present in 138 patients (15.3%) and associated with a lower 10-year patient survival rate (65.7% *vs*. 75.5%, *P* < 0.003). Similarly, 10-year graft survival was lower with elevated TCC; 56.9% *vs*. 67.3% (*P* < 0.002). Graft survival was also lower when censored for death; 81.5% *vs*. 87.3% (*P* = 0.04). In multivariable Cox analyses, impaired patient survival was significantly associated with elevated TCC [hazard ratio (HR) 1.40 (1.02–1.91), *P* = 0.04] along with male sex, recipient and donor age, smoking, diabetes, and overall survival more than 1 year in renal replacement therapy prior to engraftment. Likewise, elevated TCC was independently associated with graft loss [HR 1.40 (1.06–1.85), *P* = 0.02] along with the same covariates. Finally, elevated TCC was in addition independently associated with death-censored graft loss [HR 1.69 (1.06–2.71), *P* = 0.03] as were also HLA-DR mismatches and higher immunological risk.

**Conclusions:**

Early complement activation, assessed by plasma TCC, was associated with impaired long-term patient and graft survival.

## Introduction

Along with major improvements in posttransplant management, there has been an improvement in kidney graft survival after year 2000 (1). This is more pronounced in the long-term than in the short-term after transplantation. Still, the major reason for graft loss is caused by chronic tissue damage, as interstitial fibrosis and tubular atrophy (IF/TA), where complement activation may serve as a mediator. More knowledge on this process is needed to further improve long-term outcomes after successful kidney transplantation.

After transplantation, the innate immune system is activated by ischemia–reperfusion injury through damage-associated molecular patterns (DAMPs) released by injured cells. DAMPs are recognized by pattern recognition molecules that are present as receptors of innate immune cells either as Toll-like receptors or as soluble molecules such as upstream complement proteins (2). This activation is independent of allotype differences. In addition, if donor-specific antibodies (DSAs) are already present in the recipient at the time of transplantation, acute antibody-mediated tissue injury may be triggered by the adaptive immune system but mediated by complement activation through antibody-mediated activation of the classical pathway. Both mechanisms of complement system activation have implications for long-term outcome (3). Prolonged complement system activation is predictive of cardiovascular events, especially in diabetes and kidney failure patients undergoing dialysis (4–6). Molecules involved in the activation of the complement system have also been associated with all-cause mortality and death due to infections and cardiovascular disease in renal transplant recipients (7, 8). Thus, the role of complement in kidney transplantation is complex and multifaceted (9).

**Figure 1:**
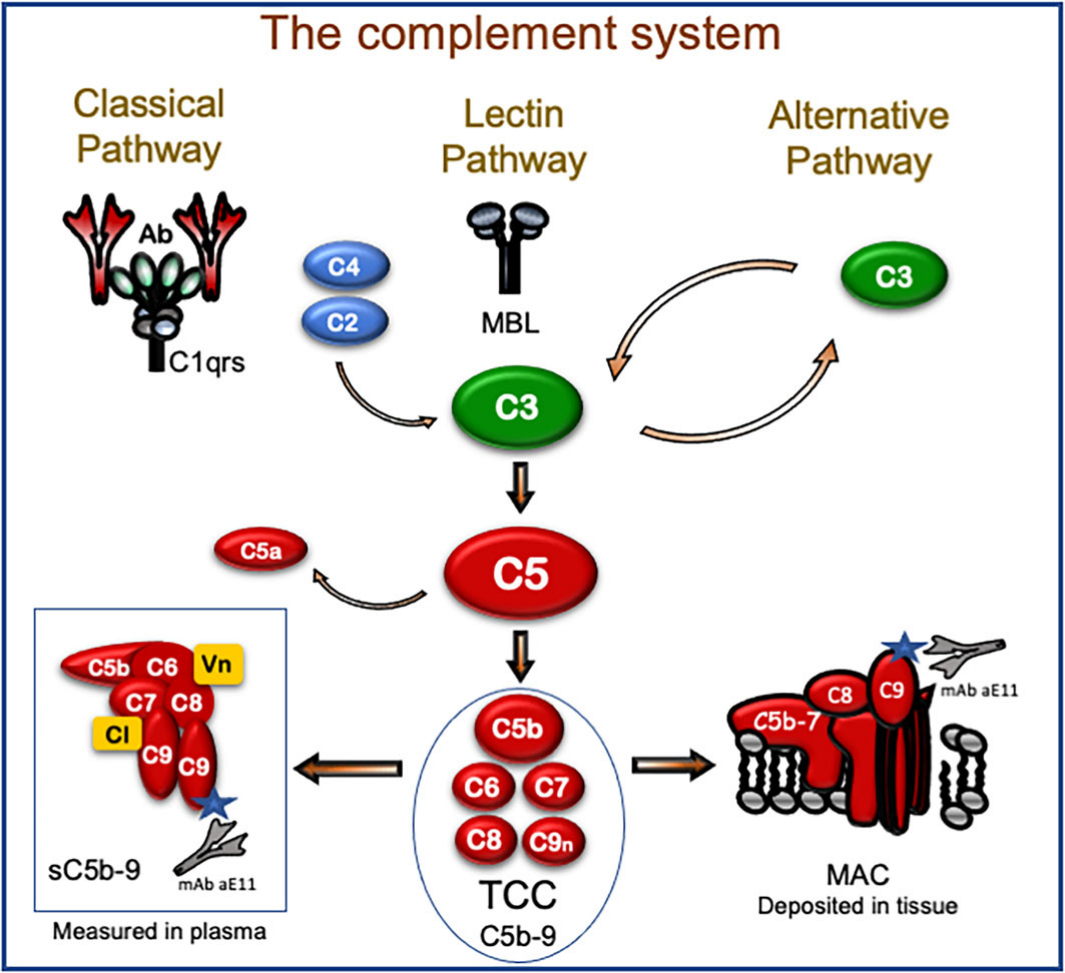
The complement system with focus on the terminal C5b-9 complement complex (TCC). Initial activation occurs through the classical, lectin, and alternative pathways that all converge on C3 that further activate C5 by cleavage into the potent proinflammatory fragment C5a and C5b. The latter initiates the formation of the TCC. TCC is a macromolecule composed of C5b, C6, C7, C8, and a couple of C9 molecules. If this assembly occurs at a surface of a lipid membrane like a microbe or cell, the macromolecule gets amphiphilic after C7 assembly and attaches to the lipid membrane. This is followed by binding of C8 and C9 within the membrane, and the complete assembly product (C5b-9) penetrates the lipid membrane and may lead to lysis. This form of TCC is frequently termed membrane attack complex (MAC) and is a pure physiochemical penetration of a lipid membrane based on molecular changes (lower right). If TCC assembles in the fluid phase (lower left), like in plasma with no lipid membrane close to the activation, a fluid-phase analog is formed. Two regulatory proteins (Vn, vitronectin; Cl, clusterin; marked yellow in the box bottom left) are necessary to cover the lipophilic sites of this macromolecule, also termed soluble TCC (sC5b-9). TCC can be detected in plasma and other body fluids and is the most frequently used complement activation product to evaluate the degree of complement activation *in vivo*. This complex is detected in ELISA, taking advantage of a monoclonal antibody (aE11) highly specific for a neoepitope exposed in C9 only when C9 is incorporated in TCC (both sC5b-9 and MAC, see bottom left and right). Thus, MAC, which is an important pathogenic factor of complement activation, inducing both inflammation and lysis, can be detected in the tissue by immunohistochemistry using the C9 neoepitope-specific antibody, whereas the TCC, which is inert and harmless, in contrast to MAC, can be detected by the same antibody in plasma using ELISA. Most importantly, TCC is a surrogate marker for C5a, which is a highly pathogenic mediator. C5a is, however, more difficult to detect *in vivo*, since it binds to the C5a receptors and has a very short half-life of 1 min, whereas TCC has a half-life of 1 h and is a very stable activation product also *in vitro*.

The complement system plays an important role in the pathophysiology of human diseases, and therapeutic inhibition of complement is rapidly approaching the clinic (10). Initial activation of complement can occur through three different pathways (**Figure 1**). The classical pathway is typically triggered by antibodies, e.g., DSA, and acute-phase proteins like C-reactive protein (CRP). The lectin pathway is initiated by recognition molecules like mannose-binding lectins, ficolins, and collectins, activated by DAMPs. Finally, the alternative pathway mainly acts as an amplification loop of the previous two pathways, increasing the activation by 80%–90% (11). All pathways converge on the level of C3, which is cleaved to the anaphylatoxin C3a and forms the C5-convertase. The latter cleaves C5 into the potent anaphylatoxin C5a, and C5b, which binds C6-C9 to form the terminal C5b-9 complement complex (TCC). TCC exists in two forms: (i) the membrane C5b-9 attack complex (MAC), which is inserted into lipid membranes, e.g., a microbe or a cell, and either leads to lysis of the microbe or cell or induces inflammation in sub-lytic doses; (ii) soluble TCC (sC5b-9) that is formed in the fluid phase, principally in the same way as MAC, but regulatory proteins are attached to the complex to render it hydrophilic enough to be soluble in plasma and other body fluids. TCC is the most stable of the complement activation products (12) and was used in the present study as a biomarker for complement activation.

Acute rejection due to DSA is dependent on systemic complement activation with positive short-term effects of complement inhibition (eculizumab) (13–15), but these effects are not seen in long-term follow-up (16). Long-term graft loss due to chronic tissue injury and scarring has, however, been postulated to be dependent on renal complement production and activation (17–20). The presence of urinary TCC (21) at 1 year after transplantation as well as C5 and C3 polymorphisms (22, 23) is associated with impaired renal function. It has further been hypothesized that the complement system might not be mandatory for maintaining the chronic kidney inflammation process leading to chronic tissue injury and scarring, as initial complement activation may lead to increased cellular infiltration, starting a self-fueling mechanism (24). It is unknown if systemic complement activation is present after the initial posttransplant phase, how it affects timing of chronic graft loss, if it is associated with cardiovascular and/or infectious events and overall survival of kidney transplant recipients, and if it is feasible to assess systemic TCC during posttransplant follow-up.

We aimed to investigate if early posttransplant complement system activation, measured as TCC in a single blood sample, was associated with decreased long-term kidney graft and overall survival in 900 consecutive kidney transplant recipients. Furthermore, the predictive ability of TCC for cause of death was investigated as a secondary outcome.

## Methods

### Patients

All kidney transplants in Norway are performed at the Oslo University Hospital–Rikshospitalet. Patients were followed at the transplant center for up to 3 months. Before transfer to their local nephrology unit, an in-depth investigation including blood sample biobanking was performed in a stable phase without ongoing infection/acute rejection episodes (the Diagnostic and Treatment biobank “*Nyrefysiologisk laboratorium*”, Biobank no. 266-2005-142234). Long-term outcomes were monitored on a national level in the Norwegian Renal Registry, a consent-based national medical quality registry with >99.9% individual coverage.

The study was approved by the Regional Medical and Health Research Committee South-East Norway (number: 2014/455). Written informed consent was obtained from all patients before any data and biological material were included in the Norwegian Renal Registry and the biobank. Other analyses from the study have been presented previously (25–27). Included in this analysis were 900 consecutively transplanted adult kidney recipients in the period October 15, 2007, to October 18, 2012, from each of whom a 10-week blood sample was biobanked. Observation period was from time of kidney transplantation to graft loss, death, or censoring date, February 24, 2020.

### Immunosuppressive Therapy

All standard immunological risk patients received basiliximab and methylprednisolone induction therapy. Patients with DSA at time of transplantation, or receiving an ABO-incompatible transplant, i.e., immunologically high-risk patients, also received intravenous human immunoglobulin and rituximab pretreatment, while panel-reactive antigen (PRA)-positive (≥20%) patients received short-course antithymocyte globulin instead of basiliximab. Standard maintenance immunosuppression was a combination of a calcineurin inhibitor (CNI), mycophenolate, and prednisolone. During the study period, the preferred CNI therapy changed, according to new center preferences in January 2011 from tacrolimus (Tac) in patients younger than 50 years and cyclosporine (CsA) in older patients and those with high body mass index (BMI) (>30 kg/m^2^) or impaired glucose tolerance to Tac for all patients except those with impaired glucose tolerance.

Therapeutic drug monitoring was performed for CNI therapy, aiming for low trough concentrations according to the Symphony study (28) for the immunological standard-risk patients (Tac: 3–7 µg/L, CsA: 75–125 µg/L from day of transplantation). For high-risk patients, target trough levels were tapered to 6–10 µg/L (Tac) and 100–150 µg/L (CsA) by 10 weeks after transplantation. Twice daily 750 mg mycophenolate mofetil (540 mg mycophenolate sodium) was used in combination with Tac and 1,000 mg (720 mg) in combination with CsA. Mycophenolate dose adjustments were performed in case of side effects. Prednisolone was initiated with 80 mg at time of transplantation and tapered according to a fixed schedule to 10 mg at 10 weeks and maintenance of 5 mg/day after month 6.

### Complement Activation Measurement

EDTA blood samples were centrifuged for 10 min at 1,800 × g within 1 h from sampling, and decanted plasma was stored at -70°C until analysis. Plasma (200 µl) was used for TCC measurement, principally as described originally in 1985 using a monoclonal antibody (aE11) as a highly specific antibody against activated C9, exposed only in C9 incorporated TCC (29). The first TCC ELISA assay was designed using this antibody as capture antibody (30). The assay has later been improved, still using aE11 as capture antibody but changing the detection to a biotinylated monoclonal antibody [mAb 9C4 (31)] against human C6, and the assay was completed with avidin–peroxidase. Results are given in complement arbitrary units (CAU)/ml according to an international standard, which set threshold for complement activation as TCC ≥0.7 CAU/ml (12).

### Endpoints

Graft loss (continuous dialysis or retransplantation) and death are reported continuously to the Norwegian Renal Registry from the locally treating nephrology centers. Estimated glomerular filtration rate (eGFR) was calculated by the MDRD-4 (modification of diet in renal disease, 4 variable) formula (32).

### Statistical Analyses

All continuous data were checked for normal distribution and presented as mean (SD) or median [interquartile range (IQR)], as appropriate. Mann–Whitney U test was applied for group comparison of independent samples. Pearson’s chi-square test was used to compare frequencies between two groups. Fisher’s exact test was used where expected cell count was less than 5. Survival rates (patient, uncensored graft, and death-censored graft) are presented as Kaplan–Meier actuarial survival estimates. Log-rank test was used to compare survival rates. Censoring date was February 24, 2020. Multivariable Cox regression models are based on backward stepwise selection, with statistical significance assessed by the Wald test. Included covariates were recipient age, male gender, diabetes at time of transplant, active smoking, renal replacement therapy (RRT) before transplant (1–12 months, 13–24 months, and above 24 months), deceased/living donor, cold ischemia time ≥14 h, cytomegalovirus positive to negative serostatus, any HLA-DR mismatch, higher immunological risk (PRA positivity or DSA positivity or incompatible ABO transplant or more than two previous kidney transplants), and TCC ≥0.7 CAU/ml at week 10 posttransplant. All covariates were check for the proportional hazards assumption and also for correlations with TCC values. Recipient age and time in RRT showed significant correlation with TCC levels but with low correlation coefficients (0.25 and 0.09, respectively) and low linear regression betas (0.0029 per year of age and 0.00053 per month in RRT, respectively). Restricted cubic spline plots for the final multivariable models are shown in **Supplementary Figure S1**. Clinical sensitivity and specificity for TCC as a biomarker for survival outcomes were performed by receiver operating characteristic (ROC) curve analyses, assessing specificity and sensitivity for the chosen cut point of 0.7 CAU/ml. Statistical analyses were performed using IBM SPSS Statistics version 26 (IBM Corp., Armonk, NY, USA) software or R (*cmprsk*-package and Gray’s test for the competing risk analysis of death-cause analysis).

## Results

### Patients

During the inclusion period, 1,317 adult kidney transplantations were performed in Norway, and 900 patients were included for this analysis. Exclusion causes were graft loss before the 10-week investigation (n = 22), understaffing at the Laboratory for Renal Physiology (n = 176), and other causes of not attending the 10-week investigation such as medical condition or early transfer to local nephrology units due to disabilities (n = 219). Demographics of the 417 patients not included were similar to the 900 included patients, but they had more often received a deceased donor kidney (*P* < 0.0001) and experienced a higher frequency of deaths during the follow-up period (*P* = 0.0005).

The cohort of patients investigated was divided according to the measured TCC level 10 weeks after transplantation: 138 patients with high plasma TCC (≥0.7 CAU/ml) *vs*. the other 762 patients (TCC <0.7 CAU/ml) (**Table 1**). Patients with high TCC were older (and hence more often treated with CsA than Tac), more frequently male, had a longer period of RRT before the transplantation and were more often retransplanted (**Table 1**).

**Table 1:** Demographics of 900 kidney transplant recipients according to TCC ≥0.7 CAU/ml (n = 138, 15.3%) and TCC <0.7 CAU/ml (n = 762, 84.7%).

Demographics	Total N = 900	TCC <0.7 N = 762	TCC ≥0.7 N = 138	*P*-value
Age, years (IQR)	55.5 (42.9–64.3)	55.0 (42.4–64.1)	58.3 (48.9–70.0)	**0.005**
Male sex, no. (%)	610 (67.8)	502 (65.9)	108 (78.3)	**0.004**
Diabetes, no. (%)	150 (16.7)	133 (17.5)	17 (12.3)	0.14
Current smoker at Tx, no. (%)	161 (17.9)	140 (18.4)	21 (15.2)	0.37
Months RRT before Tx (IQR)	12.0 (0.3–32)	11.0 (0.0–31.0)	16.5 (3.0–40.5)	**0.01**
Months RRT before Tx for non-preemptive only (n=676) (IQR)	19.5 (9.0-41.0)	19.0 (8.8-40.0)	22.5 (11.8-87.8)	**0.02**
Preemptive transplantation, no. (%)	224 (24.9)	196 (25.7)	28 (20.3)	0.17
Retransplanted, no. (%)	137 (15.2)	103 (13.5)	34 (24.6)	**0.001**
Deceased donor, no. (%)	591 (65.7)	499 (65.5)	92 (66.7)	0.79
Donor age, years (IQR)	52.0 (42.0–61.0)	52.0 (41.0–61.0)	54.0 (44.8–63)	0.08
Donor beyond 60 years, no. (%)	265 (29.4)	221 (29.0)	44 (31.9)	0.49
CMV positive to negative, no. (%)	154 (17.1)	128 (16.8)	26 (18.8)	0.56
Cold ischemia time, h (IQR) ^a^	10.2 (3.7–15.3)	10.2 (3.7–15.4)	10.2 (3.7–14.6)	0.31
Cold ischemia time ≥14 h, no. (%)	297 (33.0)	259 (34.0)	38 (27.5)	0.06
Higher immunological risk^b^(%)	117 (13.0)	100 (11.1)	17 (12.3)	0.80
PRA positive, no. (%)	51 (5.7)	46 (6.0)	5 (3.6)	0.26
DSA positive, no. (%)	58 (6.4)	52 (6.8)	6 (4.3)	0.28
ABO–incompatible, no. (%)	20 (2.2)	17 (2.2)	3 (2.2)	>0.99
>2 prior kidney transplants	20 (2.2)	14 (1.8)	6 (4.4)	0.11
HLA DR mismatch (1 or 2), no. (%)	528 (58.7)	448 (58.8)	76 (55.1)	0.42
Tacrolimus, no. (%)	435 (48.3)	392 (51.4)	43 (31.2)	**0.001**
Cyclosporine, no. (%)	457 (50.8)	365 (47.9)	92 (66.7)	**0.001**

All continuous data have a nonparametric distribution, presented as median (IQR).

Mann–Whitney U test for comparison of continuous data as all data have nonparametric distribution.

Pearson chi-square test for all dichotomous data, Fisher’s exact test where expected cell count is less than 5.

a Cold ischemia time in deceased donor transplants only (n = 591).

b Any of the following: PRA-positive, DSA-positive (2009–2012), ABO-incompatible transplant, or more than two prior kidney transplants.

CMV, cytomegalovirus; DSA, donor-specific antibody; IQR, interquartile range; PRA, panel-reactive antigen; TCC, terminal C5b-9 complement complex; Tx, transplantation; HLA DR, Human leukocyte antigen DR.

Bold values indicate significant variables.

### Patient and Graft Survival According to Plasma Terminal C5b-9 Complement Complex

Ten weeks after transplantation, median plasma TCC in the normal-range group (<0.7 CAU/ml) was 0.41 CAU/ml (range 0.12–0.69, IQR 0.33–0.50) and in the high-range group 0.87 CAU/ml (range 0.70–5.86, IQR 0.78–1.09). Renal function was not different between groups; median (IQR) eGFR in the normal-range group was 55 (43–66) ml/min/1.73 m^2^ compared to 52 (40–63) ml/min/1.73 m^2^ in the high-range group (*P* = 0.12).

The median observation time for patient survival was 9.4 years (IQR 7.7–10.6, range 0.3–12.4 years) and for graft survival 9.1 years (IQR 7.3–10.5, range 0.3–12.4 years). In the observation period, 208 patients (23.1%) died and 97 patients (10.8%) experienced isolated graft loss.

**Figure 2:**
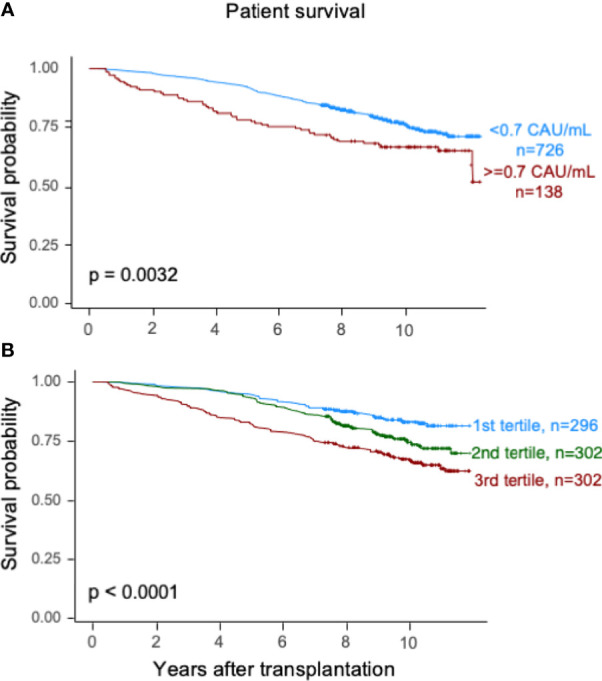
Kaplan–Meier estimates of *overall patient survival* after kidney transplantation according to plasma terminal C5b-9 complement complex (TCC) concentration 10 weeks after transplantation; **(A)** cut point of <0.7 CAU/ml and **(B)** tertiles.

**Figure 3:**
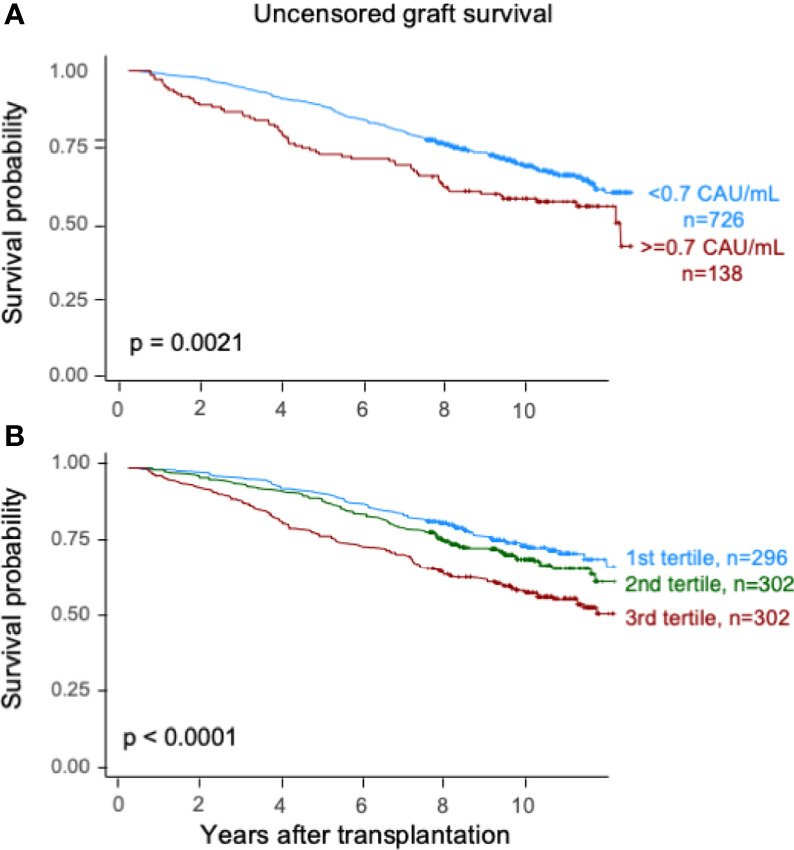
Kaplan–Meier estimates of *uncensored graft survival* after kidney transplantation according to plasma terminal C5b-9 complement complex (TCC) concentration 10 weeks after transplantation; **(A)** cut point of <0.7 CAU/ml and **(B)** tertiles.

**Figure 4:**
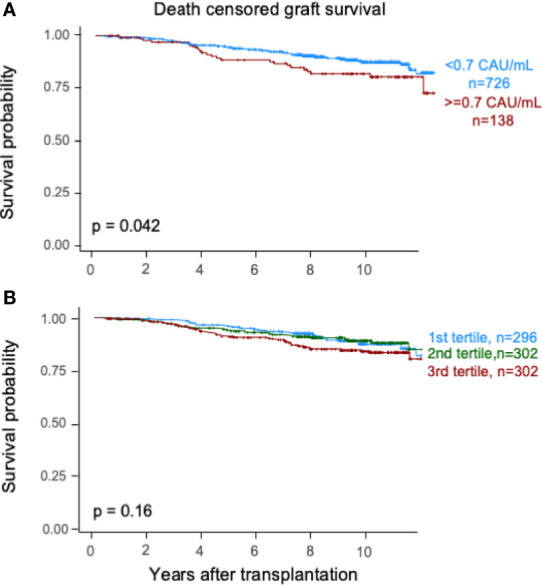
Kaplan–Meier estimates of *death-censored graft survival* after kidney transplantation according to plasma terminal C5b-9 complement complex (TCC) concentration 10 weeks after transplantation; **(A)** cut point of <0.7 CAU/ml and **(B)** tertiles.

Elevated TCC (≥0.7 CAU/ml) was associated with impaired patient survival (*P* < 0.003) with a 10-year survival rate of 65.7% (95% CI 57.7%–73.7%) compared with 75.5% (95% CI 72.2%–78.8%) (**Figure 2A**). Similarly, the graft survival uncensored for death was shorter in the high-range group compared with the normal-range group (*P* < 0.002), with 10-year graft survival rates of 56.9% (95% CI 48.7%–65.1%) and 67.3% (95% CI 63.8%–70.8%), respectively (**Figure 3A**). Also, the 10-year death-censored graft survival was shorter: 81.5% (95% CI 74.2%–88.8%) and 87.3% (95% CI 84.6%–90.0%) in the respective groups (*P* = 0.04; **Figure 4A**).

**Figure 5:**
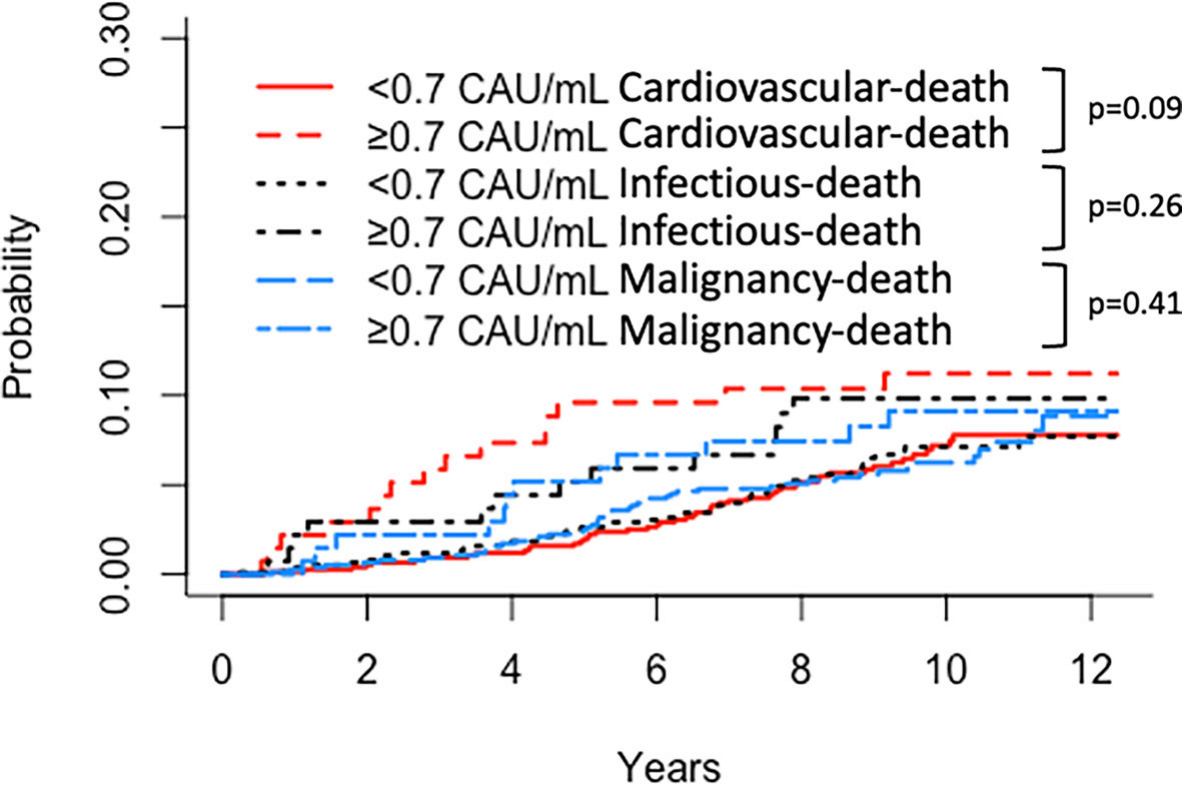
Kaplan–Meier estimates of (Red) cardiovascular, (Black) infectious, and (Blue) malignancy *patient survival* after kidney transplantation according to plasma terminal C5b-9 complement complex (TCC) concentration 10 weeks after transplantation; cut point of <0.7 CAU/ml.

Similar results were obtained for patient survival, uncensored graft survival, and death-censored graft survival when comparing TCC tertiles: 0.12–0.37, 0.38–0.52, and 0.53–5.86 CAU/ml, respectively (**Figures 2B**, **3B**, **4B**). Cause of death from cardiovascular disease, infection, and malignancy did not show significant compliance with overall patient death, although a trend (*P* = 0.09) was observed for cardiovascular disease between the two TCC groups (**Figure 5**).

### Cox Regression Models for Patient and Graft Survival

Univariate and multivariable Cox regression models for patient survival are shown in **Table 2**. Elevated TCC was independently associated with mortality. Other significant factors were recipient age and donor age over 60 years, diabetes at time of transplantation, time in RRT before transplantation (>12 months), and current smoking.

**Table 2:** Univariable and multivariable Cox regression models with TCC for patient survival after kidney transplantation 2007–2012, n = 900.

Explanatory Variable	Univariable analyses HR (95% CI)	*P*-value	Final Multivariable Model^a^, HR (95% CI)	*P*-value
Recipient age, years	1.09 (1.07–1.10)	**<0.001**	1.08 (1.07–1.10)	**<0.001**
Male sex	1.15 (0.87–1.52)	0.33		
Diabetes at time of Tx	1.88 (1.39–2.53)	**<0.001**	1.51 (1.11–2.05)	**0.008**
Smoking at time of Tx	1.73 (1.29–2.31)	**<0.001**	1.79 (1.33–2.41)	**<0.001**
RRT before Tx 0 months		**<0.001**		**<0.001**
RRT before Tx 1–12 months	1.12 (0.73–1.73)	0.60	1.38 (0.90–2.13)	0.14
RRT before Tx 13–24 months	2.30 (1.52–3.48)	**<0.001**	1.60 (1.06–2.44)	**0.03**
RRT before Tx >24 months	2.29 (1.57–3.33)	**<0.001**	2.20 (1.50–3.21)	**<0.001**
Deceased donor	3.15 (2.23–4.47)	**<0.001**		
Donor beyond 60 years	3.30 (2.55–4.27)	**<0.001**	1.80 (1.37–2.37)	**<0.001**
Cold ischemia time ≥14 h	1.64 (1.26–2.12)	**<0.001**		
CMV positive to negative status	0.88 (0.62–1.25)	0.48		
HLA DR mismatch (1 or 2)	1.01 (0.78–1.31)	0.96		
Higher immunological risk^b^	0.76 (0.50–1.16)	0.21		
TCC ≥0.7 CAU/ml	1.60 (1.17–2.18)	**0.003**	1.40 (1.02–1.91)	**0.04**

a Multivariable backward Wald Cox regression, chi-square 260.1, df = 8, P < 0.001.

b Any of the following: panel-reactive antigen positivity, donor-specific antibody positivity (2009–2012), ABO-incompatible transplantation, more than two prior kidney transplants.

CMV, cytomegalovirus; RRT, renal replacement therapy; TCC, terminal C5b-9 complement complex; Tx, transplantation; HLA DR, Human leukocyte antigen DR.

Bold values indicate significant variables.

Univariate and multivariable Cox regression models for overall (uncensored) graft survival are shown in **Table 3**. Elevated TCC was independently associated with reduced overall graft survival. The same variables that were associated with patient survival were also associated with uncensored graft loss, except for diabetes. Time in RRT was only a risk factor for uncensored graft loss if performed for more than 2 years before transplantation.

**Table 3:** Univariable and multivariable Cox regression models with TCC for overall (uncensored) graft survival after kidney transplantation 2007–2012, n = 900.

Explanatory Variable	Univariable analyses HR (95% CI)	*P*-value	Final Multivariable Model^a^, HR (95% CI)	*P*-value
Recipient age, years	1.04 (1.03–1.05)	**<0.001**	1.03 (1.02–1.05)	**<0.001**
Male sex	1.25 (0.97–1.60)	0.08		
Diabetes at time of Tx	1.52 (1.15–1.99)	**0.003**		
Smoking at time of Tx	1.70 (1.31–2.20)	**<0.001**	1.66 (1.28–2.16)	**<0.001**
RRT before Tx 0 months		**<0.001**		**0.001**
RRT before Tx 1–12 months	1.29 (0.91–1.84)	0.16	1.35 (0.95–1.93)	0.10
RRT before Tx 13–24 months	1.81 (1.25–2.61)	**0.002**	1.35 (0.93–1.96)	0.12
RRT before Tx >24 months	2.14 (1.55–2.95)	**<0.001**	1.93 (1.38–2.69)	**<0.001**
Deceased donor	2.05 (1.57–2.68)	**<0.001**	1.30 (0.96–1.75)	0.09
Donor beyond 60 years	2.45 (1.96–3.07)	**<0.001**	1.68 (1.32–2.15)	**<0.001**
Cold ischemia time ≥14 h	1.41 (1.13–1.79)	**0.003**		
CMV positive to negative status	1.01 (0.75–1.36)	0.94		
HLA DR mismatch (1 or 2)	1.07 (0.86–1.35)	0.55	1.24 (0.98–1.57)	0.07
Higher immunological risk^b^	1.13 (0.82–1.56)	0.47		
TCC ≥0.7 CAU/ml	1.54 (1.17–2.03)	**0.002**	1.40 (1.06–1.85)	**0.02**

a Multivariable backward Wald Cox regression, chi-square 161.5, df = 9, P < 0.001.

b Any of the following: panel-reactive antigen positivity, donor-specific antibody positivity (2009–2012), ABO-incompatible transplantation, more than two prior kidney transplants.

CMV, cytomegalovirus; RRT, renal replacement therapy; TCC, terminal C5b-9 complement complex; Tx, transplantation; HLA DR, Human leukocyte antigen DR.

Bold values indicate significant variables.

Univariate and multivariable Cox regression models for death-censored graft survival (isolated graft loss) are shown in **Table 4**. Elevated TCC was independently associated with impaired death-censored graft survival. Increased risk for isolated graft loss included recipient age, donor age over 60 years, male sex, deceased donor, any HLA-DR mismatch, and immunological high-risk profile as independent factors. Diabetes, time in RRT before transplantation, and current smoking were not risk factors for death-censored graft loss.

**Table 4:** Univariable and multivariable Cox regression models with TCC for death-censored graft survival after kidney transplantation 2007–2012, n = 900.

Explanatory Variable	Univariable analyses HR (95% CI)	*P*-value	Final Multivariable Model^a^, HR (95% CI)	*P*-value
Recipient age, years	0.99 (0.97–1.00)	**0.03**	0.98 (0.97–0.99)	**0.005**
Male sex	1.59 (1.02–2.47)	**0.04**	1.65 (1.05–2.59)	**0.03**
Diabetes at time of Tx	1.20 (0.73–1.96)	0.48		
Smoking at time of Tx	1.41 (0.90–2.23)	0.13		
RRT before Tx 0 months		0.06		
RRT before Tx 1–12 months	1.34 (0.79–2.30)	0.28		
RRT before Tx 13–24 months	0.83 (0.41–1.67)	0.60		
RRT before Tx >24 months	1.74 (1.05–2.89)	**0.03**		
Deceased donor	1.22 (0.82–1.82)	0.34	1.59 (1.03–2.46)	**0.04**
Donor beyond 60 years	1.42 (0.95–2.12)	0.09	1.58 (1.03–2.43)	**0.04**
Cold ischemia time ≥14 h	1.14 (0.76–1.69)	0.53		
CMV positive to negative status	1.01 (0.75–1.36)	0.94		
HLA DR mismatch (1 or 2)	1.57 (1.05–2.35)	**0.03**	1.64 (1.08–2.48)	**0.02**
Higher immunological risk^b^	1.95 (1.24–3.07)	**0.004**	2.12 (1.33–3.37)	**0.002**
TCC ≥0.7 CAU/ml	1.61 (1.01–2.55)	**0.04**	1.69 (1.06–2.71)	**0.03**

a Multivariable backward Wald Cox regression, chi-square 35.4, df = 7, P < 0.001.

b Any of the following: panel-reactive antigen positivity, donor-specific antibody positivity (2009–2012), ABO-incompatible transplantation, more than two prior kidney transplants.

CMV, cytomegalovirus; RRT, renal replacement therapy; TCC, terminal C5b-9 complement complex; Tx, transplantation; HLA DR, Human leukocyte antigen DR.

Bold values indicate significant variables.

Sensitivity analyses of the three Cox regression models when also adjusting for eGFR revealed that TCC remained a significant independent factor for all three outcome measures (SDC, Results **Supplementary Tables 1**–**3**).

### Sensitivity and Specificity of Terminal C5b-9 Complement Complex as Biomarker

**Figure 6:**
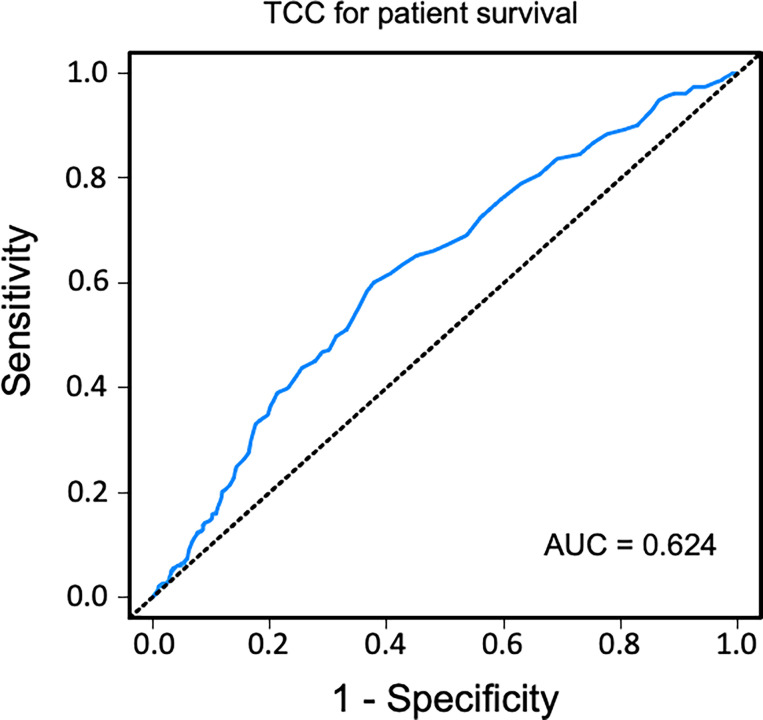
Receiver operating characteristic (ROC) curve analysis of plasma terminal C5b-9 complement complex (TCC) concentration, 10 weeks after transplantation, as a biomarker of *overall patient survival.* A cutoff value of <0.7 CAU/ml resulted in a specificity of 0.87 and sensitivity of 0.22.

**Figure 7:**
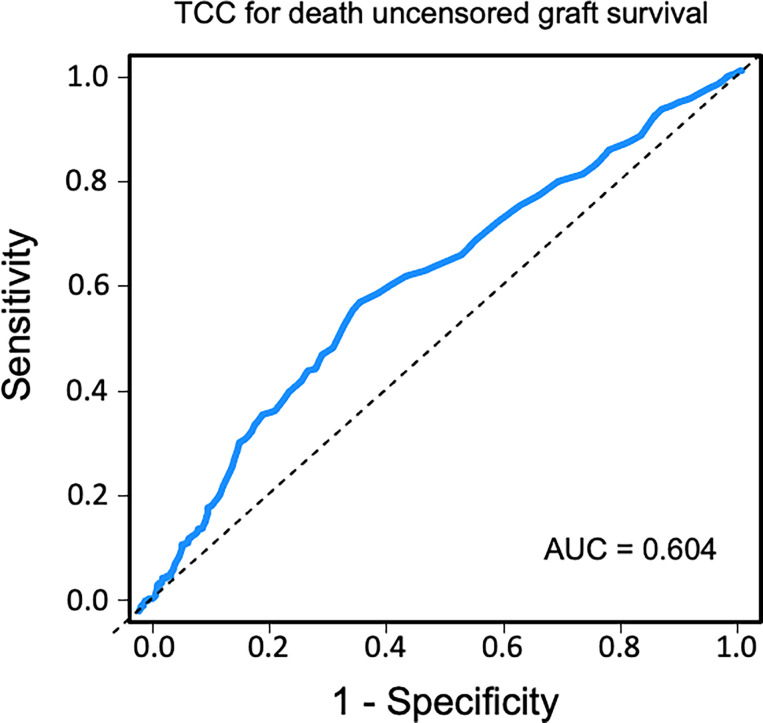
Receiver operating characteristic (ROC) curve analysis of plasma terminal C5b-9 complement complex (TCC) concentration, 10 weeks after transplantation, as a biomarker of uncensored graft survival.

**Figure 8:**
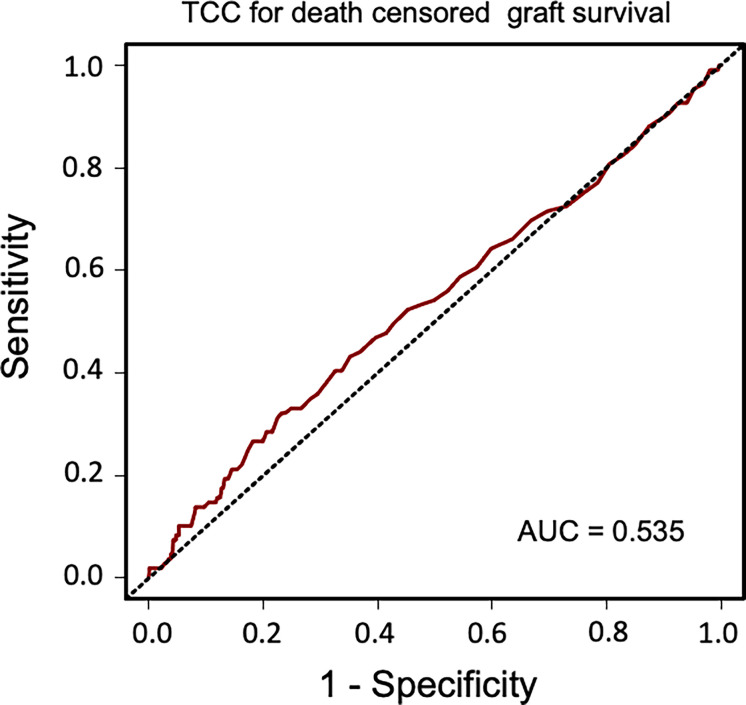
Receiver operating characteristic (ROC) curve analysis of plasma terminal C5b-9 complement complex (TCC) concentration, 10 weeks after transplantation, as a biomarker of death-censored graft survival.

Sensitivity and specificity of TCC as a clinical biomarker for overall patient survival using a ROC curve analysis showed an AUC of 0.624 (95% CI 0.583–0.665) (**Figure 6**). The specificity was 0.87 for a TCC cutoff level of 0.7 CAU/ml, with an accompanying sensitivity of 0.22. Similar analyses for uncensored and death-censored graft survival, using TCC as a biomarker, showed AUC of 0.604 (95% CI 0.565–0.644) and 0.535 (95% CI 0.475–0.596), respectively (**Figures 7**, **8**).

## Discussion

This study demonstrated that complement activation, measured as plasma TCC in a stable phase 10 weeks after kidney transplantation, was significantly associated with reduced long-term patient and graft survival. These novel findings point toward an important role of the complement system for long-term outcomes, moving it beyond the acknowledged pathophysiological effects and efficient complement inhibition treatment in acute humoral rejection (33).

Activation of the complement system in several kidney diseases suggests that this part of the innate immune system has a critical role in the pathophysiology of renal damage. Despite increased understanding of the role of complement system in renal transplantation, renal inflammaging is poorly understood. Inflammaging is a systemic chronic proinflammatory status and is a risk factor for multiple chronic diseases including chronic kidney disease (34). The histological features of naturally kidney aging are also observed in transplant injured kidneys, including IF/TA. This process may even start before donation. Previous studies have found increased systemic levels of TCC in transplantation with deceased donors but not in living donors and have linked this to a higher rejection rate (35). Once the acute ischemia–reperfusion injury experienced during transplantation is over, the complement system normalizes in most recipients. Continuous low-grade proinflammation triggering the complement system may play a crucial role for further tissue effects. TCC is a robust marker of complement activation and implies a complete activation of the complement system. In this study, plasma concentration over 0.7 CAU/ml was associated with impaired long-term patient and graft outcomes. Although the absolute value can be debated, we also confirmed our results by grouping patients into tertiles of plasma TCC values. Using ROC curve analyses, the cutoff value of 0.7 CAU/ml showed a low sensitivity but a high specificity for long-term patient survival outcome.

### Patient Survival

The hazard ratio for death associated with elevated TCC concentration was meaningful and significant even when adjusted for acknowledged factors such as age, smoking, diabetes, and time in RRT. Obviously, an association to premature death may not be uniform with respect to the cause of death. In our analysis of cause-specific mortality, high TCC indicates the strongest impact on cardiovascular deaths, although not significantly different as was also the case for deaths from infections and malignancies. These results are in line with a previous long-term follow-up study on kidney transplant patients reporting an association of increased collectin kidney 1 (a recognition molecule for the lectin pathway) with increased cardiovascular mortality (7). The same authors also reported an association of decreased Map44 levels (an inhibitory effector molecule of the lectin pathway) with increased infectious disease mortality (8), and that a high level of ficolin-3, a receptor of the complement lectin pathway, at the time of transplantation was associated with late graft loss (36). These studies focused on initial events of the lectin pathway and not on global complement activation, as assessed by TCC in the present study. However, even though complement activation predicted premature death in kidney transplant recipients in the present study, a potential cause–effect needs to be verified in interventional studies inhibiting the complement system.

### Graft Survival

Global complement activation is probably necessary to elicit a transplant rejection episode. The overall loss of kidney grafts was significantly higher in recipients with elevated TCC. This was found for both the composite end point of graft loss including death with a functioning graft as well as for death-censored graft losses. The isolated graft losses were also, in addition to TCC, independently associated with any HLA-DR mismatch, immunological high risk, and donor kidney status. Thus, patients positive for the abovementioned covariates are easily identifiable and could form a subgroup of kidney transplant patients suitable for future interventional studies evaluating complement inhibition and long-term graft survival.

A previous study staining for C4d and C3 in protocol kidney graft biopsies, early posttransplant (1 week), revealed that local complement deposition is uneventful when only the proximal C4d fragment is found but associated with graft loss when it proceeds to C3 deposits (37). Similarly, intrarenal increase of a number of complement transcripts has been associated with decreased graft function and survival (38). In this study, it appears that plasma TCC, a marker of global complement activation, independently predicted long-term graft loss. This implies that systemic complement activation is present, although the initial activation takes place locally in the transplanted kidney.

The pathophysiological mechanisms of immunologically driven IF/TA are complex and not fully explored. Preclinical studies have indicated that late graft losses are dependent on renal complement component production and activation (17, 18), consistent with the postulation that complement promotes intrarenal inflammation (24). Fibrosis is the main component of IF/TA (39, 40), but a significant association between the degree of initial renal inflammation, the BANFF i-score, and systemic inflammation has also been described (41). All these observations support the present findings of complement activation, shown to be a major mediator of kidney fibrosis through the C5a/C5aR1 axis (42), and thereby act as a key factor in development of late kidney graft losses. The findings extend the role of complement involvement beyond the common view that complement solely is involved in antibody-mediated rejection.

*Strengths and limitations*. Strengths of the study are the large number of patients and a blood sample taken at a standardized time after transplantation, after the immediate complement activation occurred during ischemia–reperfusion with acute and transient release of TCC (43). There was minimal selection of patients, limited to clinical events that prevented blood sampling, and more than two-thirds were included with none lost to follow-up. This ensures proper generalizability of the results. Furthermore, the whole material was analyzed in one batch, avoiding interassay variation over years, and with previously non-thawed plasma. A possible concern could be the different storage time of plasma in the freezer. However, using TCC as the marker of complement activation is a strength and an advantage since TCC reflects activation of the whole cascade and thus indirectly indicates release of all upstream products including the highly potent proinflammatory C5a. C5a has a short half-life of 1 min (44), rendering it more difficult to detect than TCC with a half-life of 1 h (45). Furthermore, TCC is the most stable of all activation products as we have previously reported that plasma stored for 3 years at -70°C reveals the same TCC results as when analyzed fresh in non-stored plasma (46), later also shown to be the case for samples stored for 10 years, and it is resistant to freeze–thawing up to 10 times (12). A weakness is that only one single plasma sample was obtained in this protocol, preventing us from studying the sequence profile of complement activation. An additional limitation of the study is the low sensitivity for TCC in predicting patient survival (0.22); the specificity was however substantially higher (0.87). Despite the limitations, the fact that increased TCC was significantly associated with both patient and graft survival, the latter also when censored for death, supports our main conclusions. While pretransplant donor-specific anti-HLA antibodies (DSAs) are included in the “high immunological risk” variable, dnDSA levels were not available in our database and the specific effects of dnDSAs on complement activation should be studied in the future.

In conclusion, the present study showed that complement activation measured as elevated plasma TCC in an early phase after kidney transplantation predicted premature death and long-term graft loss. Whether the complement activation actually is the cause of these adverse outcomes remains to be elucidated and should lead to trials investigating complement inhibition as a therapeutic approach for these patients.

## Data Availability Statement

The datasets analyzed in this study can be obtained from the corresponding author, who must apply for permission according to Norwegian law.

## Ethics Statement

The studies involving human participants were reviewed and approved by Regional Committee of Medical Research Ethics Health Region South-East. The patients/participants provided their written informed consent to participate in this study.

## Author Contributions

BW, SP, AH, AÅ, and TM participated in research design. AR, KM, KH, TJ, AH, and AÅ collected patient data. JL and TM performed TCC analyses. BW and AÅ performed data analysis. BW, SP, AH, AÅ, and TM wrote the paper. All authors contributed to the article and approved the submitted version.

## Conflict of Interest

The authors declare that the research was conducted in the absence of any commercial or financial relationships that could be construed as a potential conflict of interest.

## Publisher’s Note

All claims expressed in this article are solely those of the authors and do not necessarily represent those of their affiliated organizations, or those of the publisher, the editors and the reviewers. Any product that may be evaluated in this article, or claim that may be made by its manufacturer, is not guaranteed or endorsed by the publisher.

## References

[1] Coemans M Susal C Dohler B Anglicheau D Giral M Bestard O. Analyses of the Short- and Long-Term Graft Survival After Kidney Transplantation in Europe Between 1986 and 2015. Kidney Int (2018) 94:964–73. doi: 10.1016/j.kint.2018.05.018 30049474

[2] Todd JL Palmer SM. Danger Signals in Regulating the Immune Response to Solid Organ Transplantation. J Clin Invest (2017) 127:2464–72. doi: 10.1172/JCI90594 PMC5490743 28530643

[3] Sheen JH Heeger PS. Effects of Complement Activation on Allograft Injury. Curr Opin Organ Transplant (2015) 20:468–75. doi: 10.1097/MOT.0000000000000216 PMC4510836 26132735

[4] Mellbin LG Bjerre M Thiel S Hansen TK. Complement Activation and Prognosis in Patients With Type 2 Diabetes and Myocardial Infarction: A Report From the DIGAMI 2 Trial. Diabetes Care (2012) 35:911–7. doi: 10.2337/dc11-1642 PMC3308270 22357179

[5] Speidl WS Exner M Amighi J Kastl SP Zorn G Maurer G. Complement Component C5a Predicts Future Cardiovascular Events in Patients With Advanced Atherosclerosis. Eur Heart J (2005) 26:2294–9. doi: 10.1093/eurheartj/ehi339 15917276

[6] Lines SW Richardson VR Thomas B Dunn EJ Wright MJ Carter AM. Complement and Cardiovascular Disease–The Missing Link in Haemodialysis Patients. Nephron (2016) 132:5–14. doi: 10.1159/000442426 26695077

[7] Smedbraten J Sagedal S Asberg A Hartmann A Rollag H Mjoen G. Collectin Liver 1 and Collectin Kidney 1 of the Lectin Complement Pathway Are Associated With Mortality After Kidney Transplantation. Am J Transplant (2017) 17:265–71. doi: 10.1111/ajt.13933 27341702

[8] Smedbraten J Mjoen G Hartmann A Asberg A Rollag H Mollnes TE. Low Level of MAp44, an Inhibitor of the Lectin Complement Pathway, and Long-Term Graft and Patient Survival; A Cohort Study of 382 Kidney Recipients. BMC Nephrol (2016) 17:148. doi: 10.1186/s12882-016-0373-9 27760523 PMC5070230

[9] Biglarnia AR Huber-Lang M Mohlin C Ekdahl KN Nilsson B. The Multifaceted Role of Complement in Kidney Transplantation. Nat Rev Nephrol (2018) 14:767–81. doi: 10.1038/s41581-018-0071-x 30367174

[10] Garred P Tenner AJ Mollnes TE. Therapeutic Targeting of the Complement System: From Rare Diseases to Pandemics. Pharmacol Rev (2021) 73:792–827. doi: 10.1124/pharmrev.120.000072 33687995 PMC7956994

[11] Harboe M Mollnes TE. The Alternative Complement Pathway Revisited. J Cell Mol Med (2008) 12:1074–84. doi: 10.1111/j.1582-4934.2008.00350.x PMC3865650 18419792

[12] Bergseth G Ludviksen JK Kirschfink M Giclas PC Nilsson B Mollnes TE. An International Serum Standard for Application in Assays to Detect Human Complement Activation Products. Mol Immunol (2013) 56:232–9. doi: 10.1016/j.molimm.2013.05.221 23787367

[13] Glotz D Russ G Rostaing L Legendre C Tufveson G Chadban S. Safety and Efficacy of Eculizumab for the Prevention of Antibody-Mediated Rejection After Deceased-Donor Kidney Transplantation in Patients With Preformed Donor-Specific Antibodies. Am J Transplant (2019) 19:2865–75. doi: 10.1111/ajt.15397 PMC9328661 31012541

[14] Marks WH Mamode N Montgomery RA Stegall MD Ratner LE Cornell LD. Safety and Efficacy of Eculizumab in the Prevention of Antibody-Mediated Rejection in Living-Donor Kidney Transplant Recipients Requiring Desensitization Therapy: A Randomized Trial. Am J Transplant (2019) 19:2876–88. doi: 10.1111/ajt.15364 PMC6790671 30887675

[15] Ricklin D Barratt-Due A Mollnes TE. Complement in Clinical Medicine: Clinical Trials, Case Reports and Therapy Monitoring. Mol Immunol (2017) 89:10–21. doi: 10.1016/j.molimm.2017.05.013 28576323

[16] Schinstock CA Bentall AJ Smith BH Cornell LD Everly M Gandhi MJ. Long-Term Outcomes of Eculizumab-Treated Positive Crossmatch Recipients: Allograft Survival, Histologic Findings, and Natural History of the Donor-Specific Antibodies. Am J Transplant (2019) 19:1671–83. doi: 10.1111/ajt.15175 PMC6509017 30412654

[17] Sheerin NS Risley P Abe K Tang Z Wong W Lin T. Synthesis of Complement Protein C3 in the Kidney Is an Important Mediator of Local Tissue Injury. FASEB J (2008) 22:1065–72. doi: 10.1096/fj.07-8719com 18039928

[18] Curci C Castellano G Stasi A Divella C Loverre A Gigante M. Endothelial-To-Mesenchymal Transition and Renal Fibrosis in Ischaemia/Reperfusion Injury Are Mediated by Complement Anaphylatoxins and Akt Pathway. Nephrol Dial Transplant (2014) 29:799–808. doi: 10.1093/ndt/gft516 24463188

[19] Pratt JR Basheer SA Sacks SH. Local Synthesis of Complement Component C3 Regulates Acute Renal Transplant Rejection. Nat Med (2002) 8:582–7. doi: 10.1038/nm0602-582 12042808

[20] Brown KM Kondeatis E Vaughan RW Kon SP Farmer CK Taylor JD. Influence of Donor C3 Allotype on Late Renal-Transplantation Outcome. N Engl J Med (2006) 354:2014–23. doi: 10.1056/NEJMoa052825 16687714

[21] Lammerts RGM Eisenga MF Alyami M Daha MR Seelen MA Pol RA. Urinary Properdin and Sc5b-9 Are Independently Associated With Increased Risk for Graft Failure in Renal Transplant Recipients. Front Immunol (2019) 10:2511. doi: 10.3389/fimmu.2019.02511 31736953 PMC6830301

[22] Jeong JC Hwang YH Kim H Ro H Park HC Kim YJ. Association of Complement 5 Genetic Polymorphism With Renal Allograft Outcomes in Korea. Nephrol Dial Transplant (2011) 26:3378–85. doi: 10.1093/ndt/gfr025 21393613

[23] Varagunam M Yaqoob MM Dohler B Opelz G. C3 Polymorphisms and Allograft Outcome in Renal Transplantation. N Engl J Med (2009) 360:874–80. doi: 10.1056/NEJMoa0801861 19246358

[24] Stegall MD Chedid MF Cornell LD. The Role of Complement in Antibody-Mediated Rejection in Kidney Transplantation. Nat Rev Nephrol (2012) 8:670–8. doi: 10.1038/nrneph.2012.212 23026942

[25] Dahle DO Asberg A Hartmann A Holdaas H Bachtler M Jenssen TG. Serum Calcification Propensity Is a Strong and Independent Determinant of Cardiac and All-Cause Mortality in Kidney Transplant Recipients. Am J Transplant (2016) 16:204–12. doi: 10.1111/ajt.13443 26375609

[26] Thorsen IS Bleskestad IH Asberg A Hartmann A Skadberg O Brede C. Vitamin D as a Risk Factor for Patient Survival After Kidney Transplantation: A Prospective Observational Cohort Study. Clin Transplant (2019) 33:e13517. doi: 10.1111/ctr.13517 30844090

[27] Heldal TF Ueland T Jenssen T Hartmann A Reisaeter AV Aukrust P. Inflammatory and Related Biomarkers Are Associated With Post-Transplant Diabetes Mellitus in Kidney Recipients: A Retrospective Study. Transpl Int (2018) 31:510–9. doi: 10.1111/tri.13116 29341300

[28] Ekberg H Tedesco-Silva H Demirbas A Vitko S Nashan B Gurkan A. Reduced Exposure to Calcineurin Inhibitors in Renal Transplantation. N Engl J Med (2007) 357:2562–75. doi: 10.1056/NEJMoa067411 18094377

[29] Mollnes TE Lea T Harboe M Tschopp J. Monoclonal Antibodies Recognizing a Neoantigen of Poly(C9) Detect the Human Terminal Complement Complex in Tissue and Plasma. Scand J Immunol (1985) 22:183–95. doi: 10.1111/j.1365-3083.1985.tb01870.x 4035298

[30] Mollnes TE Lea T Froland SS Harboe M. Quantification of the Terminal Complement Complex in Human Plasma by an Enzyme-Linked Immunosorbent Assay Based on Monoclonal Antibodies Against a Neoantigen of the Complex. Scand J Immunol (1985) 22:197–202. doi: 10.1111/j.1365-3083.1985.tb01871.x 2412280

[31] Mollnes TE Redl H Hogasen K Bengtsson A Garred P Speilberg L. Complement Activation in Septic Baboons Detected by Neoepitope-Specific Assays for C3b/iC3b/C3c, C5a and the Terminal C5b-9 Complement Complex (TCC). Clin Exp Immunol (1993) 91:295–300. doi: 10.1111/j.1365-2249.1993.tb05898.x 7679061 PMC1554676

[32] Levey AS Coresh J Greene T Stevens LA Zhang YL Hendriksen S. Using Standardized Serum Creatinine Values in the Modification of Diet in Renal Disease Study Equation for Estimating Glomerular Filtration Rate. Ann Intern Med (2006) 145:247–54. doi: 10.7326/0003-4819-145-4-200608150-00004 16908915

[33] Tan EK Bentall A Dean PG Shaheen MF Stegall MD Schinstock CA. Use of Eculizumab for Active Antibody-Mediated Rejection That Occurs Early Post-Kidney Transplantation: A Consecutive Series of 15 Cases. Transplantation (2019) 103:2397–404. doi: 10.1097/TP.0000000000002639 PMC6699919 30801549

[34] Franceschi C Garagnani P Parini P Giuliani C Santoro A. Inflammaging: A New Immune-Metabolic Viewpoint for Age-Related Diseases. Nat Rev Endocrinol (2018) 14:576–90. doi: 10.1038/s41574-018-0059-4 30046148

[35] Cravedi P Heeger PS. Complement as a Multifaceted Modulator of Kidney Transplant Injury. J Clin Invest (2014) 124:2348–54. doi: 10.1172/JCI72273 PMC4038571 24892709

[36] Smedbraten YV Sagedal S Mjoen G Hartmann A Fagerland MW Rollag H. High Ficolin-3 Level at the Time of Transplantation Is an Independent Risk Factor for Graft Loss in Kidney Transplant Recipients. Transplantation (2015) 99:791–6. doi: 10.1097/TP.0000000000000422 25222012

[37] Sund S Hovig T Reisaeter AV Scott H Bentdal O Mollnes TE. Complement Activation in Early Protocol Kidney Graft Biopsies After Living-Donor Transplantation. Transplantation (2003) 75:1204–13. doi: 10.1097/01.TP.0000062835.30165.2C 12717204

[38] Cernoch M Hruba P Kollar M Mrazova P Stranavova L Lodererova A. Intrarenal Complement System Transcripts in Chronic Antibody-Mediated Rejection and Recurrent IgA Nephropathy in Kidney Transplantation. Front Immunol (2018) 9:2310. doi: 10.3389/fimmu.2018.02310 30356754 PMC6189372

[39] Nankivell BJ Shingde M Keung KL Fung CL Borrows RJ O'Connell PJ. The Causes, Significance and Consequences of Inflammatory Fibrosis in Kidney Transplantation: The Banff I-IFTA Lesion. Am J Transplant (2018) 18:364–76. doi: 10.1111/ajt.14609 29194971

[40] Garcia-Carro C Dorje C Asberg A Midtvedt K Scott H Reinholt FP. Inflammation in Early Kidney Allograft Surveillance Biopsies With and Without Associated Tubulointerstitial Chronic Damage as a Predictor of Fibrosis Progression and Development of *De Novo* Donor Specific Antibodies. Transplantation (2017) 101:1410–5. doi: 10.1097/TP.0000000000001216 27163535

[41] Garcia-Carro C Dorje C Asberg A Midtvedt K Scott H Reinholt FP. Kidney Allograft Subclinical Rejection Modulates Systemic Inflammation Measured by C-Reactive Protein at 1 Year After Transplantation. Clin Transplant (2018) 32:e13196. doi: 10.1111/ctr.13196 29380890

[42] Peng Q Wu W Wu KY Cao B Qiang C Li K. The C5a/C5aR1 Axis Promotes Progression of Renal Tubulointerstitial Fibrosis in a Mouse Model of Renal Ischemia/Reperfusion Injury. Kidney Int (2019) 96:117–28. doi: 10.1016/j.kint.2019.01.039 31029505

[43] de Vries DK van der Pol P van Anken GE van Gijlswijk DJ Damman J Lindeman JH. Acute But Transient Release of Terminal Complement Complex After Reperfusion in Clinical Kidney Transplantation. Transplantation (2013) 95:816–20. doi: 10.1097/TP.0b013e31827e31c9 23348894

[44] Wagner JL Hugli TE. Radioimmunoassay for Anaphylatoxins: A Sensitive Method for Determining Complement Activation Products in Biological Fluids. Anal Biochem (1984) 136:75–88. doi: 10.1016/0003-2697(84)90308-7 6711816

[45] Mollnes TE. Early- and Late-Phase Activation of Complement Evaluated by Plasma Levels of C3d,g and the Terminal Complement Complex. Complement (1985) 2:156–64. doi: 10.1159/000467856 3878764

[46] Mollnes TE Garred P Bergseth G. Effect of Time, Temperature and Anticoagulants on *In Vitro* Complement Activation: Consequences for Collection and Preservation of Samples to be Examined for Complement Activation. Clin Exp Immunol (1988) 73:484–8. PMC1541764 2463123

