# Supplementary material for: Elevated Terminal C5b-9 Complement Complex 10 Weeks Post Kidney Transplantation Was Associated With Reduced Long-Term Patient and Kidney Graft Survival
Source: Front Immunol. 2021 Oct 25;12:738927. doi: 10.3389/fimmu.2021.738927 (PMC8573334; doi:10.3389/fimmu.2021.738927)
Supplement: Supplementary file 1 [file DataSheet_1.pdf]

Supplementary Material

1 SUPPLEMENTARY TABLES

SDC, Results Table S1 – including eGFR. Uni- and multivariable Cox regression models with TCC for patient survival after kidney transplantation 2007-2012, n=900.

**Explanatory Variable**	**Univariable analyses**	**P-**	**Final Multivariable**	**P-**
Recipient age, years	1.09 (1.07-1.10)	**<0.001**	1.08 (1.07-1.10)	**<0.001**
Male sex	1.15 (0.87-1.52)	0.33		
Diabetes at time of Tx	1.88 (1.39-2.53)	**<0.001**	1.51 (1.11-2.05)	**0.008**
Smoking at time of Tx	1.73 (1.29-2.31)	**<0.001**	1.79 (1.33-2.41)	**<0.001**
RRT[b] before Tx 0 months		**<0.001**		**<0.001**
RRT before Tx 1-12 months	1.12 (0.73-1.73)	0.60	1.38 (0.90-2.13)	0.14
RRT before Tx 13-24 months	2.30 (1.52-3.48)	**<0.001**	1.60 (1.06-2.44)	**0.03**
RRT before Tx >24 months	2.29 (1.57-3.33)	**<0.001**	2.20 (1.50-3.21)	**<0.001**
Deceased donor	3.15 (2.23-4.47)	**<0.001**		
Donor beyond 60 years	3.30 (2.55-4.27)	**<0.001**	1.80 (1.37-2.37)	**<0.001**
Cold ischemia time ≥14 hours	1.64 (1.26-2.12)	**<0.001**		
eGFR MDRD[c]	0.98 (0.97-0.99)	**<0.001**		
CMV pos to neg status	0.88 (0.62-1.25)	0.48		
HLA DR mismatch (1 or 2)	1.01 (0.78-1.31)	0.96		
Higher immunological risk[d]	0.76 (0.50-1.16)	0.21		
TCC ≥0.7 CAU/mL	1.60 (1.17-2.18)	**0.003**	1.40 (1.02-1.91)	**0.04**

[a] Multivariable backward Wald Cox regression, Chi-Square 260.1, df=8, p<0.001

[b] Renal Replacement Therapy

[c] Estimated Glomerular Filtration Rate at 8-weeks posttransplant based on Modification of Diet in Renal Disease formula

[d] Any of the following: Panel Reactive Antigen positivity, Donor Specific Antibody positivity (2009-2012), ABO incompatible transplantation, more than two prior kidney transplants

SDC, Results Table Table S2 – including eGFR. Uni- and multivariable Cox regression models with TCC for overall graft survival after kidney transplantation 2007-2012, n=900.

Explanatory Variable	Univariable	P-	Final Multivariable	P-
Recipient age, years	1.04 (1.03-1.05)	**<0.001**	1.03 (1.02-1.04)	**<0.001**
Male sex	1.25 (0.97-1.60)	0.08		
Diabetes at time of Tx	1.52 (1.15-1.99)	**0.003**		
Smoking at time of Tx	1.70 (1.31-2.20)	**<0.001**	1.65 (1.27-2.14)	**<0.001**
RRT[b] before Tx 0 months		**<0.001**		**0.001**
RRT before Tx 1-12 months	1.29 (0.91-1.84)	0.16	1.36 (0.95-1.93)	0.09
RRT before Tx 13-24 months	1.81 (1.25-2.61)	**0.002**	1.44 (1.00-2.08)	0.05
RRT before Tx >24 months	2.14 (1.55-2.95)	**<0.001**	2.07 (1.50-2.86)	**<0.001**
Deceased donor	2.05 (1.57-2.68)	**<0.001**	1.30 (0.96-1.75)	0.09
Donor beyond 60 years	2.45 (1.96-3.07)	**<0.001**	1.68 (1.32-2.15)	**<0.001**
Cold ischemia time ≥14 hours	1.41 (1.13-1.79)	**0.003**		
eGFR MDRD[c]	0.98 (0.97-0.99)	**<0.001**	0.99 (0.99-1.00)	0.06
CMV pos to neg status	1.01 (0.75-1.36)	0.94		
HLA DR mismatch (1 or 2)	1.07 (0.86-1.35)	0.55	1.24 (0.98-1.57)	0.07
Higher immunological risk[d]	1.13 (0.82-1.56)	0.47		
TCC ≥0.7 CAU/mL	1.54 (1.17-2.03)	**0.002**	1.34 (1.01-1.77)	**0.04**

[a] Multivariable backward Wald Cox regression, Chi-Square 161.5, df=9, p<0.001

[b] Renal Replacement Therapy

[c] Estimated Glomerular Filtration Rate at 8-weeks posttransplant based on Modification of Diet in Renal Disease formula

[d] Any of the following: Panel Reactive Antigen positivity, Donor Specific Antibody positivity (2009-2012), ABO incompatible transplantation, more than two prior kidney transplants

SDC, Results Table Table S3 – including eGFR. Uni- and multivariable Cox regression models with TCC for death censored graft survival after kidney transplantation 2007-2012, n=900.

Explanatory Variable	Univariable	P-	Final Multivariable	P-
Recipient age, years	0.99 (0.97-1.00)	**0.03**	0.97 (0.96-0.98)	**<0.001**
Male sex	1.59 (1.02-2.47)	**0.04**	1.63 (1.04-2.55)	**0.03**
Diabetes at time of Tx	1.20 (0.73-1.96)	0.48		
Smoking at time of Tx	1.41 (0.90-2.23)	0.13	1.56 (0.99-2.46)	0.06
RRT[b] before Tx 0 months		0.06		
RRT before Tx 1-12 months	1.34 (0.79-2.30)	0.28		
RRT before Tx 13-24 months	0.83 (0.41-1.67)	0.60		
RRT before Tx >24 months	1.74 (1.05-2.89)	**0.03**		
Deceased donor	1.22 (0.82-1.82)	0.34	1.47 (0.95-2.27)	0.09
Donor beyond 60 years	1.42 (0.95-2.12)	0.09	1.58 (1.03-2.43)	**0.04**
Cold ischemia time ≥14 hours	1.14 (0.76-1.69)	0.53		
eGFR MDRD[c]	0.97 (0.96-0.99)	**<0.001**	0.97 (0.95-0.98)	**<0.001**
CMV pos to neg status	1.01 (0.75-1.36)	0.94		
HLA DR mismatch (1 or 2)	1.57 (1.05-2.35)	**0.03**	1.71 (1.12-2.59)	**0.01**
Higher immunological risk[d]	1.95 (1.24-3.07)	**0.004**	1.92 (1.21-3.06)	**0.006**
TCC ≥0.7 CAU/mL	1.61 (1.01-2.55)	**0.04**	1.67 (1.04-2.68)	**0.04**

[a] Multivariable backward Wald Cox regression, Chi-Square 57.6, df=8, p<0.001

[b] Renal Replacement Therapy

[c] Estimated Glomerular Filtration Rate at 8-weeks posttransplant based on Modification of Diet in Renal Disease formula

[d] Any of the following: Panel Reactive Antigen positivity, Donor Specific Antibody positivity (2009-2012), ABO incompatible transplantation, more than two prior kidney transplants

2 SUPPLEMENTARY FIGURE

Supplementary Figure 1.

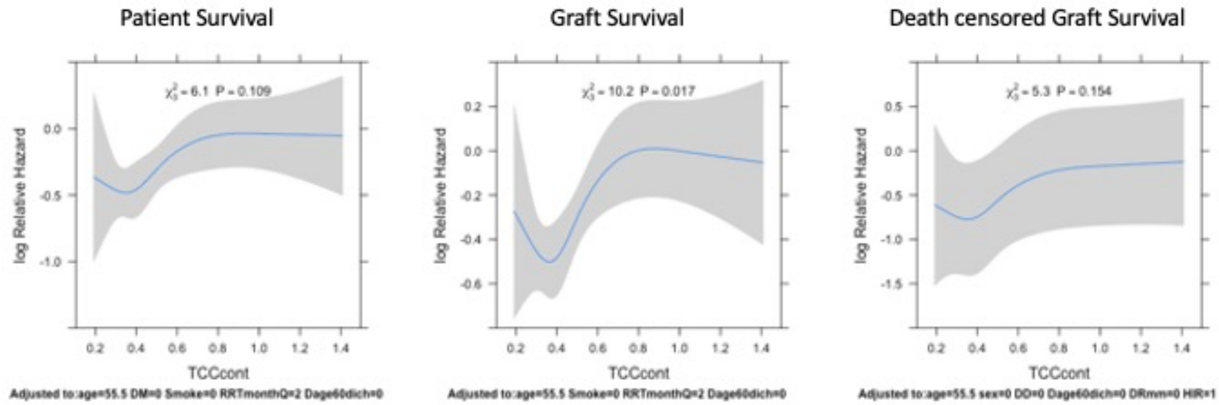

SDC, Results, Figure S1. Restricted cubic spline plots of the final multivariable models for (from left) patient-, graft- and death censored graft survival with TCC levels as continuous variable on the x-axis and 4 knots. Plots are produced using the *survival*-package in R.
